# Draft genome sequence of the plastic-degrading fungus *Fusicolla acetilerea* strain NIBRFGC000505922

**DOI:** 10.1186/s12863-026-01408-8

**Published:** 2026-01-28

**Authors:** Munkhgerel Dalantai, Seung-Yeol Lee, Hee-Young Jung, Young-Hyun You, Changmu Kim, Seung-Yoon Oh

**Affiliations:** 1 Department of Biology and Microbiology, Changwon National University, Changwon, Republic of Korea; 2 Department of Plant Medicine, Kyungpook National University, Daegu, Republic of Korea; 3 Institute of Plant Medicine, Kyungpook National University, Daegu, Republic of Korea; 4 Species Diversity Research Division, National Institute of Biological Resources, Incheon, Republic of Korea; 5 School of Advanced Biosciences, Changwon National University, Changwon, Republic of Korea

**Keywords:** Biosynthetic gene clusters, Carbohydrate-active enzymes, *Fusicolla acetilerea*, Genome assembly, Hypocreales, Plastic degradation

## Abstract

**Objectives:**

*Fusicolla acetilerea* is a saprophytic fungus in the family Nectriaceae (order Hypocreales). A strain, NIBRFGC000505922, was isolated from rhizosphere soil in South Korea and showed plastic-degrading activity against polycaprolactone and polylactic acid, indicating its potential for treating biodegradable plastic waste. However, genomic data for *F. acetilerea* are not yet available, which limits understanding of its biodegradation abilities. The objective of this study is to present the first draft genome sequence of *F. acetilerea* strain NIBRFGC000505922 to offer insights into its gene content, including carbohydrate-active enzymes (CAZymes), biosynthetic gene clusters (BGCs), and potential plastic-degrading enzymes.

**Data description:**

We assembled the genome of *F. acetilerea* strain NIBRFGC000505922 using Illumina NovaSeq and PacBio Revio HiFi sequences. The genome sequence is 50.2 Mb across 12 scaffolds (N50: 5.2 Mb, GC: 47.6%). BUSCO analysis against Sordariomycetes_obd10 showed 97.9% completeness. We predicted 13,961 protein-coding genes and assigned functions to 10,824 of them. Among these, 466 are CAZyme and 36 are biosynthetic gene clusters; additionally, 12 candidate genes linked to plastic degradation were identified, supporting the strain’s potential for plastic breakdown. This genome resource offers a valuable foundation for understanding the molecular mechanisms underlying fungal plastic degradation and exploring its applications in environmental biotechnology.

## Objectives

*Fusicolla* Bonord. 1851 is a genus of saprophytic fungi in the family Nectriaceae (order Hypocreales), first described by Bonorden [1], and is mainly isolated from soil and decaying organic matter [2, 3]. Although less studied than its close relative *Fusarium*, the genus *Fusicolla* now includes around 22 recognized species [4]. *Fusicolla acetilerea* was first identified in 1976 as *Fusarium merismoides* var. *acetilereum* [5] and was later renamed by Gräfenhan & Seifert [2]. This species produces both macroconidia and microconidia, with the macroconidia typically being straight to slightly curved and having 3–4 septa [2, 4].

A strain NIBRFGC000505922 (= KNUF-20-PBU01) of *F. acetilerea* was isolated from rhizosphere soil in South Korea. It exhibited plastic-degrading activity against polycaprolactone (PCL) and polylactic acid (PLA), indicating its potential in treating biodegradable plastic waste [6]. Among fungal enzymes, cutinases and lipases are reported to hydrolyze synthetic polymers such as PLA, PCL, and polyethylene terephthalate (PET) [7]. These enzymes have been successfully used for degrading synthetic polymers [8]. However, genomic resources for *F. acetilerea* are still limited, restricting insights into its molecular biodegradation mechanisms. Here, we present the first draft genome sequence of *F. acetilerea* strain NIBRFGC000505922, offering new insights into its gene repertoire, including CAZymes, biosynthetic gene clusters, and putative plastic-degrading enzymes.

## Data description

The strain NIBRFGC000505922 was cultured and underwent genome sequencing and annotation. Detailed experimental and analytical procedures are provided in the Materials and Methods section (Data file 1, Table 1) [9]. A total of 396,484 PacBio HiFi reads (4.9 Gb; average length of ~ 12.4 kb; average quality Q31) were obtained for de novo assembly (Data set 1, Table 1) [10]. Additionally, Illumina NovaSeq sequencing produced 27.2 million DNA reads (4.1 GB, Q20 = 95.4%, Q30 = 87.4%) for error correction (Data set 2, Table 1) [11]. and 53.3 million RNA reads (8.0 GB, Q20 = 98.8%, Q30 = 96.4%) for transcriptome-based gene annotation (Data set 3, Table 1) [12]. The final genome assembly of *Fusicolla acetilerea* strain NIBRFGC000505922 comprised 12 scaffolds (N50 = 5.2 Mb), totaling 50.2 Mb, with an average coverage of 98.1× (Data set 4, Table 1) [13]. The GC content was 47.6%, and BUSCO analysis [14] demonstrated high completeness, with recovery rates of 99.5% (Fungi_odb10), 98.3% (Ascomycota_odb10), and 97.9% (Sordariomycetes_odb10) (Data file 2, Table 1) [15].

**Table 1:** Overview of data files/data sets

Label	Name of data file/data set	File types (file extension)	Data repository and identifier (DOI or accession number)
Data set 1	PBU01-DNA_HiFi.fastq.gz	Fastq file (.fastq.gz)	NCBI Sequence Read Archive SRR35822075 (http://identifiers.org/insdc.sra: SRR35822075) [10]
Data set 2	PBU01-DNA_1.fastq.gz PBU01-DNA_2.fastq.gz	Fastq file (.fastq.gz)	NCBI Sequence Read Archive SRR35602807 (http://identifiers.org/insdc.sra: SRR35602807) [11]
Data set 3	PBU01-RNA_1.fastq.gz PBU01-RNA_2.fastq.gz	Fastq file (.fastq.gz)	NCBI Sequence Read Archive SRR35822851 (http://identifiers.org/insdc.sra: SRR35822851) [12]
Data set 4	Genome assembly of *F. acetilerea* strain NIBRFGC000505922	Fasta contig (.fna)	NCBI GenBank Database JBQPWL000000000 (http://identifiers.org/ nucleotide: JBQPWL000000000.1) [13]
Data file 1	Materials and Methods	Document file (.docx)	Figshare (10.6084/m9.figshare.30399268.v2) [9]
Data file 2	Summary of genome assembly for *F. acetilerea* strain NIBRFGC000505922	Document file (.docx)	Figshare (10.6084/m9.figshare.30399331.v1) [15]
Data file 3	Predicted plastic-degrading enzymes identified from *F. acetilerea* strain NIBRFGC000505922	Document file (.docx)	Figshare (10.6084/m9.figshare.30399274.v1) [20]

Gene prediction of *F. acetilerea* strain NIBRFGC000505922 identified a total of 13,961 protein-coding gene models, of which 10,824 were functionally annotated using InterProScan [16]. The analysis using dbCAN3 [17] identified 466 genes encoding carbohydrate-active enzymes (CAZymes). These comprise 255 auxiliary activities (AA), 107 carbohydrate esterases (CE), 70 glycoside hydrolases (GH), 25 carbohydrate-binding modules (CBM), 20 glycosyltransferases (GT), and 8 polysaccharide lyases (PL). AntiSMASH [18] predicted a total of 36 putative biosynthetic gene clusters (BGCs), including 8 polyketide synthases (PKSs), 11 nonribosomal peptide synthetases (NRPSs), 8 terpene synthases, 1 ribosomally synthesized and post-translationally modified peptide (RiPP), 1 isocyanide-NRP cluster, and 7 hybrid clusters. Based on PlasticDB [19], a total of 12 plastic-degrading gene was detected targeting several plastics (e.g., PET, PCL, and PLA) as substrates (Data file 3, Table 1) [20]. Taken together, these genomic features provide a foundational resource for future studies on the taxonomy and diversity of *Fusicolla* and related Hypocreales.

## Limitations

We used a hybrid approach (PacBio HiFi long reads plus Illumina NovaSeq short reads), but the assembly for *F. acetilerea* strain NIBRFGC000505922 has not reached chromosome scale. A fully resolved build will likely require Hi-C data. The plastic-degrading candidates are based on bioinformatic results. Therefore, their enzyme activities and degradation performance still need bench validation under relevant substrates and conditions.

## Data Availability

The data described in this Data note can be freely and openly accessed on NCBI Sequence Read Archive under PRJNA1301341. The genome assembly is available National Center for Biotechnology Information (NCBI) at the under accession JBQPWL000000000 ( http://identifiers.org/nucleotide : JBQPWL000000000.1). Please see Table 1 and references [10–13] for details and links to the data.

## Abbreviations

AA: Auxiliary activities
BGCs: Biosynthetic gene clusters
CAZymes: Carbohydrate-active enzymes
CBM: Carbohydrate-binding modules
CE: Carbohydrate esterases
GH: Glycoside hydrolases
GT: Glycosyltransferases
NRPSs: Nonribosomal peptide synthetases
PCL: Polycaprolactone
PET: Polyethylene terephthalate
PKSs: Polyketide synthases
PLA: Polylactic acid
PL: Polysaccharide lyases
RiPP: Ribosomally synthesized and post-translationally modified peptide
